# Thermal tolerance and survival are modulated by a natural gradient of infection in differentially acclimated hosts

**DOI:** 10.1093/conphys/coae015

**Published:** 2024-04-15

**Authors:** Jérémy De Bonville, Ariane Côté, Sandra A Binning

**Affiliations:** Groupe de Recherche Interuniversitaire en Limnologie et en Environnement Aquatique (GRIL), Département de Sciences Biologiques, Université de Montréal, 1375 Av.Théres̀e-Lavoie-Roux, Montréal, QC, H2V 0B3, Canada; Groupe de Recherche Interuniversitaire en Limnologie et en Environnement Aquatique (GRIL), Département de Sciences Biologiques, Université de Montréal, 1375 Av.Théres̀e-Lavoie-Roux, Montréal, QC, H2V 0B3, Canada; Groupe de Recherche Interuniversitaire en Limnologie et en Environnement Aquatique (GRIL), Département de Sciences Biologiques, Université de Montréal, 1375 Av.Théres̀e-Lavoie-Roux, Montréal, QC, H2V 0B3, Canada

**Keywords:** cestodes, climate change, concomitant stressors, critical thermal maximum, critical thermal minimum, endoparasites, freshwater fish, trematodes

## Abstract

Wild ectotherms are exposed to multiple stressors, including parasites, that can affect their responses to environmental change. Simultaneously, unprecedented warm temperatures are being recorded worldwide, increasing both the average and maximum temperatures experienced in nature. Understanding how ectotherms, such as fishes, will react to the combined stress of parasites and higher average temperatures can help predict the impact of extreme events such as heat waves on populations. The critical thermal method (CTM), which assesses upper (CT_max_) and lower (CT_min_) thermal tolerance, is often used in acclimated ectotherms to help predict their tolerance to various temperature scenarios. Despite the widespread use of the CTM across taxa, few studies have characterized the response of naturally infected fish to extreme temperature events or how acute thermal stress affects subsequent survival. We acclimated naturally infected pumpkinseed sunfish (*Lepomis gibbosus*) to four ecologically relevant temperatures (10, 15, 20 and 25°C) and one future warming scenario (30°C) for 3 weeks before measuring CT_max_ and CT_min_. We also assessed individual survival the week following CT_max_. Parasites were counted and identified following trials to relate infection intensity to thermal tolerance and survival. Interestingly, trematode parasites causing black spot disease were negatively related to CT_max_, suggesting that heavily infected fish are less tolerant to acute warming. Moreover, fish infected with yellow grub parasites showed decreased survival in the days following CT_max_ implying that the infection load has negative survival consequences on sunfish during extreme warming events. Our findings indicate that, when combined, parasite infection and high prolonged average temperatures can affect fish thermal tolerance and survival, emphasizing the need to better understand the concomitant effects of stressors on health outcomes in wild populations. This is especially true given that some parasite species are expected to thrive in warming waters making host fish species especially at risk.

## Teaser

This study shows that different parasites may influence the thermal tolerance and the survival of their hosts, pumpkinseed sunfish, suggesting that such stressors are important to increase the ecological relevance of thermal tolerance studies of wild animals.

## Introduction

Wildlife is experiencing increases in average daily temperatures, wider daily and seasonal thermal variations and an increase in the frequency, duration and intensity of extreme weather events, such as heat waves and cold snaps (Meehl and Tebaldi, 2004; Seneviratne *et al.,* 2014; IPCC, 2022). In ectotherms, temperature directly affects the rate of physiological functions, such as enzyme activity and energy metabolism (Fry, 1971; Jutfelt *et al.,* 2024). Thus, acute changes to the thermal environment are expected to have detrimental consequences for wild ectotherms, including increases in mortality, elevated stress levels and altered immune functions (Roth *et al.,* 2010; Genin *et al.,* 2020; Jørgensen *et al.,* 2022). Although some aquatic animals are regularly exposed to rapid temperature changes, for instance, whilst crossing the thermocline during vertical migrations or due to diurnal temperature fluctuations in shallow waters or intertidal zones (Bates and Morley, 2020; Desforges *et al.,* 2023), exposure of shallow aquatic species to rapid thermal changes will likely be intensified due to increases in average surface water temperatures (IPCC, 2022). Thus, estimating the susceptibility of ectotherms to acute warming events is crucial to understand the risk posed by global climate change to wild populations.

Lower thermal tolerance is also relevant physiologically and ecologically. Rapid cooling events occur naturally due to cold snaps and marine upwelling events (Donaldson *et al.,* 2008; Lehmann and Myrberg, 2008) or anthropogenically, due to industrial runoff (Michie *et al.,* 2020), possibly leading to mass mortality events (Reid *et al.,* 2022). With climate change causing more thermal variability, sudden cold snaps could become more widespread in the wild (ELPC, 2019), highlighting the need to consider lower thermal tolerance when studying how climate change will affect wild fish populations (Reid *et al.,* 2022). Many experimental studies have attempted to determine species’ thermal limits and acclimation to rapidly changing temperatures. However, the focus on temperature as the sole stressor ignores the reality of climate change, whereby multiple stressors are expected to impact wild populations simultaneously (Pirotta *et al.,* 2022).

Indeed, wild animals experience multiple biotic and abiotic stressors concurrently, which may interact. For instance, hypoxia generally exerts antagonistic effects on upper thermal tolerance (Andreassen *et al.,* 2022), possibly due to limitation of oxygen supply to tissues at warmer temperatures, limiting aerobic scope (Ern *et al.,* 2016). However, very few studies have explored the ways that biotic stressors, such as parasite infection, interact with heat stress. Despite being ubiquitous in most natural environments, parasites are seldom considered in studies of wild animal performance (Chrétien *et al.,* 2022). Ignoring infection is a potentially serious oversight since it can lead to erroneous conclusions at individual host, population and community levels (Timi and Poulin, 2020). Furthermore, some parasites, such as helminths, are expected to thrive with increasing temperatures caused by climate change: warmer water temperatures can enhance parasite growth (Macnab and Barber, 2012) and virulence (Thomas and Blanford, 2003), making host fish populations particularly at risk (Marcogliese, 2008; but see Wood *et al.,* 2023). Parasites can impose a range of physiological costs on hosts including increasing host metabolic demands (Binning *et al.,* 2013; Thambithurai *et al.,* 2022). Furthermore, thermoreceptors in the skin allow fish to detect and react to changing temperatures through endocrine signalling (Haesemeyer, 2020). Damage to the skin caused by parasites could potentially affect how fish react to changes in temperature. Thus, infection could impair an individual’s ability to withstand acute thermal stress by constraining oxygen uptake or impairing sensorial functions. Indeed, experimental infection with Chytrid fungus (*Batrachochytrium dendrobatidis*), led to a 4°C decrease in the upper thermal tolerance of frogs (*Litoria spenceri*) (Greenspan *et al.,* 2017). Similarly, upper thermal tolerance has been negatively related to parasite load in naturally infected juvenile brown trout (*Salmo trutta*) (Bruneaux *et al.,* 2017), bluegill (*Lepomis macrochirus*) and longear sunfish (*Lepomis megalotis*) (Lutterschmidt *et al.,* 2007).

Interestingly, the impact of infection on thermal tolerance appears to be parasite species-specific. In three cyprinid species, a higher intensity of trematode metacercaria (e.g. black spot disease) did not lead to a change in upper or lower thermal tolerance (Hockett and Mundahl, 1989). Likewise, Powell and Gamperl (2016) did not find differences in the upper thermal tolerance of Atlantic cod (*Gadus morhua*) infected with microsporidia (*Loma morhua*) compared to uninfected conspecifics. The specific physiological costs of parasites on hosts could explain why results are mixed. Unfortunately, the few studies that have combined measures of parasite infection and thermal tolerance focus on infection with a single parasite species at a given temperature, without evaluating how these relationships are affected by different acclimation temperatures. Shedding light on the relationship between parasites and host thermal tolerance in the context of climate change is essential for improving our understanding of ecological consequences infection could exert on the host, from the individual to the community level.

The critical thermal method (CTM) can be used to assess upper (CT_max_) and lower (CT_min_) thermal tolerance of animals acclimated at a given temperature (Beitinger *et al.,* 2000; Morgan *et al.,* 2018). Knowing the upper and lower physiological thermal thresholds of a species can help us predict which are more at risk of mass mortality events during heat waves or cold snaps (Genin *et al.,* 2020; Reid *et al.,* 2022; Desforges *et al.,* 2023). Although CTM is meant to be a sublethal measure, researchers tend to assess survival only up to 24 h after the test (Cowan *et al.,* 2023; Stewart *et al.,* 2023). Yet, in nature, the negative effects of thermal stress can persist for days following the onset of a heating (Suryan *et al.,* 2021) or a cooling (Reid *et al.,* 2022) event. Indeed, extreme events exacerbated by global change are likely to pose a greater threat to the survival of wild populations compared to average temperature increases (Vasseur *et al.,* 2014). Thus, assessing thermal tolerance and individual survival in the days following a thermal challenge can help shed light on a species risk to climate change and improve conservation goals for aquatic ectotherms.

Here, we assessed the upper and lower thermal tolerance of a naturally infected freshwater fish (pumpkinseed sunfish; *Lepomis gibbosus* (Linnaeus 1758)) acclimated to ecologically relevant temperatures and documented their survival for 1 week following an acute thermal maximum trial (CT_max_). By working with wild-caught fish housed in a flow-through system and considering their natural gradient of infection to assess their thermal tolerance in a lab-controlled environment, we hope to increase ecological relevance of results obtained in a laboratory (Turko *et al.,* 2023). We expected acclimation temperature to affect both CT_max_ and CT_min_ and that parasite intensity would also be related to thermal tolerance. We also expected survival to be related to infection intensity and acclimation temperature. We predicted that fish with a higher parasite load would have a smaller thermal tolerance (lower CT_max_ and higher CT_min_) and that heavily infected individuals would have lower survival rates. We further predicted that the effects of parasites would be more apparent at warmer temperatures, as these concomitant stressors could interact to negatively affect outcomes (Teffer *et al.,* 2019).

## Materials and Methods

### Model species

Pumpkinseed (Family: Centrarchidae) are native to temperate and boreal lakes of eastern North America and mostly inhabit shallow vegetated littoral zones (Scott and Crossman, 1974). Summer temperatures in their native range can vary from 22 to 31°C, depending on the lake characteristics and region (Tomeček *et al.,* 2007). The CT_max_ of pumpkinseed has been estimated ~35°C for fish acclimated at 20°C and up to 39°C for those acclimated at 30°C (Becker and Genoway, 1979; Rooke *et al.,* 2017). Whilst the CT_min_ of pumpkinseed is comparatively understudied, Becker *et al.* (1977) found that bluegill, a closely related centrarchid, acclimated to 15°C had an average CT_min_ of 1.7°C.

**Figure 1:**
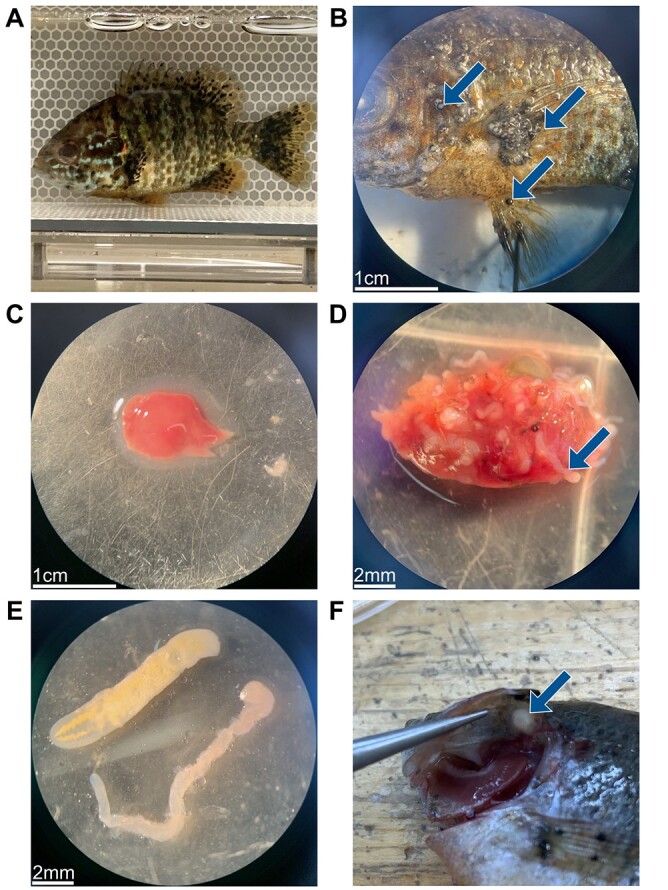
Photographs showing parasites of their host. (**A**) Black spot trematodes (*Uvulifer sp., Apophallus sp.*) on a live pumpkinseed. (**B**) Black spots shown on the body, pectoral and pelvic fins. (**C**) A fish liver with no parasites. (**D**) A fish liver infected by bass tapeworms (*P. ambloplitis*). (**E**) comparison between a yellow grub (*C*. *marginatum*) on the top left and a bass tapeworm on the bottom right. (**F**) Encysted yellow grub on the opercular flap of a pumpkinseed (F).

In the Laurentian region of Quebec where this study took place, pumpkinseed are abundant and frequently infected by trematodes causing black spot disease (*Uvulifer sp., Apophallus sp.)*, bass tapeworms (Cestoda: *Proteocephalus ambloplitis*), as well as yellow grubs (Trematoda: *Clinostomum marginatum*) (Scott and Crossman, 1974; Guitard *et al.,* 2022) (Fig. 1). Previous work in these populations has demonstrated significant relationships between metabolic rates and parasite infection at the cellular (Mélançon *et al.,* 2023) and whole-organism levels (Guitard *et al.,* 2022; Thambithurai *et al.,* 2022). The wide thermal tolerance of this species as well as high prevalence and intensities of infection make it an ideal candidate to evaluate how both factors interact in wild populations.

**Table 1:** Relationship between thermal trait and predictors as selected by the best fitting model for each trait

Response	Predictors	*d.f.*	*F*-value	*P*-value	*R* ^2^_aj_
CT_max_ (*n* = 215)	**Black spots**	1, 205	7.94	**0.005**	**0.99**
	Bass tapeworm	1, 205	0.46	0.50	
	Yellow grubs	1, 205	0.19	0.67	
	Fish mass	1, 205	3.40	0.07	
	**Treatment**	5, 205	2790.90	**<0.001**	
CT_min_ (*n* = 200)	Black spots	1, 185	0.43	0.51	0.97
	Bass tapeworms	1, 185	1.27	0.26	
	Yellow grubs	1, 185	0.21	0.65	
	Fish mass	1, 185	0.54	0.46	
	**Treatment**	5, 185	1145.01	**<0.001**	
	Fish mass * Treatment	5, 185	4.16	**0.001**	
Tolerance (*n* = 100)	Black spots	1, 93	1.99	0.38	0.26
	Bass tapeworms	1, 93	0.08	0.37	
	Yellow grubs	1, 93	0.01	0.08	
	Fish mass	1, 93	2.26	0.08	
	**Treatment**	2, 93	16.19	**<0.001**	

See Supplementary Material Table S1B for model results with estimates for each coefficient tested in the models.

Significant predictors are shown in bold.

### Fish collection and husbandry

We collected fish in 2020 and 2021 in Lake Cromwell (SBL, QC, Canada; 45.98898°N, −74.00013°W) located on the Université de Montréal’s Station de Biologie des Laurentides (SBL). Pumpkinseed were collected during two seasons, fall and summer, to assess the effects of environmentally realistic acclimation temperatures. In 2020, we collected 102 fish in September (mean ± standard deviation (SD): total length (TL) = 7.3 ± 0.9 cm, mass = 6.85 ± 2.51 g). In 2021, we collected 113 fish in June for the summer group (mean ± SD: TL = 7.7 ± 0.8 cm, mass = 7.83 ± 2.60 g) and 100 fish in September for the fall group (mean ± SD: TL = 8.1 ± 0.9 cm, mass = 9.59 ± 3.60 g). Lab conditions and treatments were the same for all fish, except for the light cycle, which followed a typical fall diel cycle (12 L:12 D) for fall fish from 2020 and 2021 and a summer cycle (14 L:10 D) for fish collected in summer 2021. Baited minnow traps with bread in mesh bags were set at different sites along the littoral zone of the lake for 1–2 h after which selected fish were collected and brought back to the lab within 30 min (400-m walk from lake to lab). Fish underwent a hydrogen peroxide dip (2.5 ml of 3% H_2_O_2_ per litre of fresh water) for 30 min, to limit the proliferation of external pathogens (Rach *et al.,* 2000). This treatment does not affect endoparasites in pumpkinseed (Guitard *et al.,* 2022). Fish were then distributed amongst three flow-through 600-l (215 × 60 × 60 cm, L × W × H) Living Stream tanks (Frigid Units, Inc.) separated into three sections and provided with artificial plants and PVC tubes for shelter. Water was pumped from the nearby Lake Croche (45.99003°N, −74.00567°W; upstream of Lake Cromwell, but with similar physicochemical properties) and filtered through a sand pool filter, oxygenated in a collecting basin and sterilized with UV light before entering the tanks. Twenty-four hours following collection, fish were weighed, measured and individually marked with coloured visible implant elastomer tags (VIE, NorthWest Marine Technology, Inc.) on either side of the dorsal fin with a 29-gauge needle. Fish were fed to satiety twice a day with frozen bloodworms (Hikari®) and tanks were siphoned daily.

### Acclimation treatments

We acclimated fish at four temperatures reflecting the natural conditions found in Lake Cromwell (10, 15, 20 and 25°C) and to one temperature representing a future climate change scenario (30°C). Historic records and colleagues (Roxane Maranger, Alice Parkes) working on Lake Cromwell were consulted in 2020 to help establish accurate acclimation temperatures. We also deployed a temperature logger (HOBO Pendant® MX2201, USA, accuracy: ±0.5°C, resolution: 0.04°C) 1 m below the surface from June 2021 to August 2022 to measure seasonal and daily variations in the littoral zone of Lake Cromwell to confirm the thermal profile experienced by these fish (Supplementary Fig. S1).

For the fall groups (2020 and 2021), fish were acclimated to 10, 15 and 20°C whilst acclimation temperatures of 20, 25 and 30°C were chosen for summer experiments (2021). In Lake Cromwell, temperatures of 25°C represent warm summer temperatures, 20°C represents lower summer temperatures, 15°C represents early fall/late spring temperatures and 10°C represents late fall/early spring (Supplementary Fig. S1). During a summer heat wave, lake temperatures can reach up to 28°C. Thus, the 30°C acclimation group represents a long thermal acclimation to heat wave temperatures to which we added 2°C to simulate a future climate change scenario (IPCC, 2022). One day following tagging, water temperature in the flow-through tanks was increased or decreased by 1–1.5°C per day, depending on treatment, until reaching the desired acclimation temperature. Temperature in the tanks was monitored twice daily with handheld thermometers, and logged through the duration of the acclimation period and experiments with a HOBO MX2201 at 10-min intervals (Supplementary Fig. S2). Temperature was maintained by controllers (Inkbird ITC-308S, China) activating either a cooling (EK20 immersion cooler, Thermo ScientificTM, USA) or a heating immersion 1500 W or 2000 W coil (GESAIL, China) to ensure the tanks remained at the desired acclimation temperature (±0.2°C). Depending on the temperature, water inflow in the tanks ranged from 4.8 to 22.8 l/h, ensuring an inflow of oxygenated water and a complete water change between 26 and 125 h, maintaining a stable temperature and high water quality throughout acclimation. Natural temperature changes in Lake Croche, where the tank water is pumped from, or power outages caused spikes in temperature during the acclimation periods (Supplementary Fig. S2A: Oct-11, C: Sep-23). Pumps (ECO-396, EcoPlus®, China) were used to circulate the water to ensure homogenous mixing, and air bubblers were deployed throughout the tank to maintain oxygen saturation.

Fish were acclimated for 3 weeks to allow thermal compensation before being tested for CT_max_ (Becker and Genoway, 1979; Dent and Lutterschmidt, 2003). A minimum of a 3-week acclimation period was also necessary before conducting trials, as it takes approximately 21 days for trematode cercaria to encyst and be visible as black spots on the fish (Berra and Au, 1978).

### **CT**_**max**_ **& CT**_**min**_ **trials**

Fish collected in fall 2020 were only tested for CT_max_, whilst fish from fall 2021 were tested for CT_min_. In summer 2021, we assessed both CT_max_ and CT_min_ for the same individuals. CT_max_ and CT_min_ trials were conducted in a test arena (93 × 42 × 44 cm) containing 50 l of water connected to a heating/cooling sump (50 × 30 × 26 cm) that contained 25 l, for a combined volume of 75 l (Supplementary Fig. S3). In each corner, there were air bubblers and an inflow or outflow of water to ensure continuous mixing and homogenous temperatures between both tanks.

Fish were fed in the morning the day before the trial, then fasted between 24–30 h before the start. Fish were habituated to the arena overnight at the same temperature as their acclimation tank, with temperature maintained stable by controllers (Inkbird ITC-308S, China). Trials were conducted the next day, starting between 09:12 and 14:47. Two to three trials were done for each acclimation group (number of fish/trial = 8–18).

Our CTM protocol followed methods described in Morgan *et al.* (2018). To begin the trial, two 1000-W immersion coil heaters (GESAIL, China) were turned on in the sump for CT_max_. For CT_min_, a cooling coil was used (EK20 immersion cooler, Thermo Scientific™, USA) as well as 1-l ice blocks. Temperature was increased or decreased following a desired rate of 0.3°C min^−1^ (mean ± SD: CT_max_ = 0.26 ± 0.04°C min^−1^, CT_min_ = −0.3 ± 0.1°C min^−1^) (For ramping curves see Supplementary Fig. S4). Similar ramping rates were used previously in CT_max_ experiments on pumpkinseed (Becker and Genoway, 1979; Dent and Lutterschmidt, 2003). Temperature was logged throughout the trials (2020 logger: HOBO® MX2203, USA, accuracy: ±0.2°C, resolution: 0.01°C; 2021 logger: HOBO® MX2303, accuracy: ±0.2°C, resolution: 0.04°C). Following methods described in Morgan *et al.* (2018), we conducted preliminary trials with 3 fish collected in 2020 in Lake Cromwell to ensure changes in fish internal temperature matched the water. Fish were anaesthetized with eugenol (1:10 mix, at 0.4 ml/l), had a thermocouple inserted in the deep dorsal muscle (Type K, RS PRO) and were exposed to a ramping rate of ~0.3°C min^−1^, which confirmed that internal ramping rates followed ramping rates in the water (Supplementary Fig. S5).

We defined loss of equilibrium (LOE) as the point when a fish could no longer maintain a stable upright position in the water column for 3 continuous seconds (Lutterschmidt and Hutchison, 1997; Morgan *et al.,* 2018). LOE for CT_min_ for fish acclimated to 10 and 15°C was slightly different. As these fish reached low temperatures and became inactive, we gently poked them with a hand net. If fish did not respond to poking for 3 seconds, we considered the fish to have reached its CT_min_ (Ford and Beitinger, 2005). To obtain the CT_max_ or CT_min_, the temperature measured by the logger at the time at which the fish was taken out was used. There was a delay in the temperature measures recorded by the logger used in 2020 (response time: 7 min), as this model was encased in a thick plastic case and was less responsive to real-time water temperature changes. Thus, for these fish (*n* = 102), values recorded from a digital thermometer (HI98509, HANNA Instruments, USA, accuracy: ±0.2°C, resolution: 0.1°C) placed in the arena with the fish during the experiments were used instead.

After reaching LOE, fish were transferred to a numbered plastic bag placed in a cooler both containing water at the same acclimation temperature of the group being tested and recovered for at least 1 h (or until they swam normally). Before being transferred back to their holding tanks, fish were identified, weighed and measured. Following trials, fish were monitored for mortality (for 24 h in fall 2020 and 2021 or over the following week in summer 2021) twice a day when fish were fed and were considered deceased when found at the bottom of the tank with an absence of opercular movements. Following survival assessment, fish either underwent a CT_min_ trial (summer 2021 group acclimated to 20, 25 and 30°C) or were sacrificed in an overdose of eugenol solution for the fall 2020 and 2021 groups (1:10 mix, at 4 ml/l) and frozen at −18°C for dissection to assess parasite load. Summer 2021 fish were sacrificed and preserved in the same way following CT_min_. Thermal tolerance was measured as the difference between CT_max_ and CT_min_, for fish that went through both trials (summer 2021).

### Parasite screening

Frozen fish were thawed to assess infection load. The fish were pinned to a Styrofoam pad by the fins and observed under a stereomicroscope to count encysted black spot metacercaria on their left side (body and fins), which was doubled to obtain the total amount for each fish. A subset of pumpkinseed (*n* = 89) sampled for another project confirmed that there is no significant difference in the number of black spots present between the left and right sides of the body (non-parametric paired samples Wilcoxon test; *P* = 0.284, Supplementary Fig. S6). The amount of black spots found on both sides of the gills was added to the total amount. All fish were infected to some extent by black spots (prevalence = 100%; median = 163 parasites; range = 6–1107). Dissection of internal organs and muscles was conducted to look for the presence of other parasites (specifically in the gills, heart, liver, digestive tract, gonads and in the abdominal cavity). We estimated the load of other endoparasites, which mainly consisted of bass tapeworm (*Proteocephalus ambloplitis)* found in the liver and digestive tract, as well as the yellow grub (*C. marginatum*) mostly found in the gills, heart and in the muscle. Whilst 94.3% of fish were infected by *P. amploblitis* (median = 15 parasites; range = 0–186), only 23.8% of fish were infected by *C*. *marginatum* (median = 0 parasites; range = 0–10) (Fig. 1 for parasite images). The prevalence values from our fish are similar to results obtained by Guitard *et al.* (2022) on the same population of ﻿similar size (mean ± SD: total length = 8.5 ± 0.7 cm, body mass = 10.24 ± 2.46 g) also sampled with minnow traps in July 2019 (black spot: 100%, bass tapeworm: 93%, yellow grub: 26%). As these parasites all have complex life cycles, they do not reproduce inside their fish host, nor transmit directly between hosts. Pumpkinseed were randomly distributed between acclimation treatments when collected, and we do not expect temperature to influence parasite load once fish were in the lab. Individual fish mass was corrected for the presence of parasites (as suggested by Timi and Poulin, 2020) by basing parasite weight averages on values measured by Guitard *et al.* (2022) on the same species.

### Statistical analyses

All data were analysed in R v.4.2.2 (http://www.R-project.org/). We used general linear models (lm function in R) to model the effect of acclimation treatment and parasite count on thermal tolerance traits (CT_max_, CT_min_ and thermal tolerance). To find the best fitted model to explain each trait, fish mass (corrected for parasites), the number of black spots, the number of bass tapeworms, the number of yellow grubs, the acclimation treatment, the interaction between mass and acclimation treatment as well as all the interactions between parasite groups and acclimation treatment were used as predictors in the three full models. Although parasite count and fish mass were correlated (cor = 0.29), collinearity of variables included in the models was tested using the variance inflation factor (VIF). As the VIF was low (<2), both variables were included in the models (Legendre and Legendre, 2012).

We used Akaike’s Information Criterion (AIC) to compare candidate models for each trait and select the most parsimonious model (Burnham *et al.,* 2011; Thambithurai *et al.,* 2022; Stewart *et al.,* 2023). Parasites were included in all tested models, but mass, acclimation and the interactions were removed in a stepwise manner. We compared general linear models using ΔAIC, AIC cumulative weights and *R*^2^, and, for the best model, the effects of each selected predictor were assessed using the Anova() function, and estimates of each predictor were evaluated using the summary() function to assess the direction of the effect, if significant. We used *post hoc* contrasts of least square means using the emmeans package (Lenth, 2023) to assess differences between acclimation temperatures. We visually assessed model assumptions with diagnostic plots, which were met for all models.

The packages ‘survival’ (Therneau, 2022) and ‘survminer’ (Kassambara *et al.,* 2021) were used for the survival analyses, following methods from (Fox and Weisberg, 2011). A proportional hazards survival regression (coxph function from the ‘survival’ package) was used to test the effect of three different parasite species and fish mass on survival following CT_max_ tests at three acclimation treatments (20, 25 and 30°C) (Cox, 1972). By using this model, we calculated the influence of covariables on survival probability over a set period of time (7 days post-CT_max_ trial). The response variable (hazard ratio, HR) represents the risk of death during the survival assessment period. Thus, an HR < 1 indicates that the associated predictor is protective (improved survival) and an HR > 1 increases the risk (decreases survival) (Cox, 1972; Clark *et al*., 2003). We tested multiple candidate models, which always included parasites, to which we added acclimation temperature, fish mass and all two-way interactions. Starting from the most complex model, we used likelihood ratio tests (LRT) to compare models and removed non-significant interactions and predictors (Supplementary Table S2) (Therneau and Grambsch, 2000; Cortese *et al.,* 2021). The effects of each predictor were assessed using the Anova() function, and hazard ratios (coefficients) of each predictor were obtained using the summary() function.

Model diagnostics were assessed for all models tested, where we checked for violation of the assumption of proportional hazards, for nonlinearity in the relationship between the log hazard and the covariates used in the models and for influential data (Fox and Weisberg, 2011). The assumptions of proportional hazard were tested using the cox.zph() and visually observed by plotting the Schoenfeld residuals with the ggcoxzph() function from the survminer package and validating that all predictors where encompassed by the 95% confidence interval lines (Supplementary Fig. S7). The martingale residuals were plotted against covariates to confirm the non-linearity assumption, and influential cases were negligeable when observing residuals of the model (all dfbetas < 1).

### Ethical declaration

Fish collection, treatment and experiments were conducted with approval from the Université de Montréal’s animal care committee (Comité de déontologie de l’expérimentation sur les animaux; certificate numbers 20–042 & 21–028) and the Ministère des Forêts, de la Faune et des Parcs (permit numbers 2020-07-10-1730-15-S-P and 2021-05-18-1833-15-S-P).

## Results

### Influence of acclimation and parasites on thermal tolerance traits

**Figure 2:**
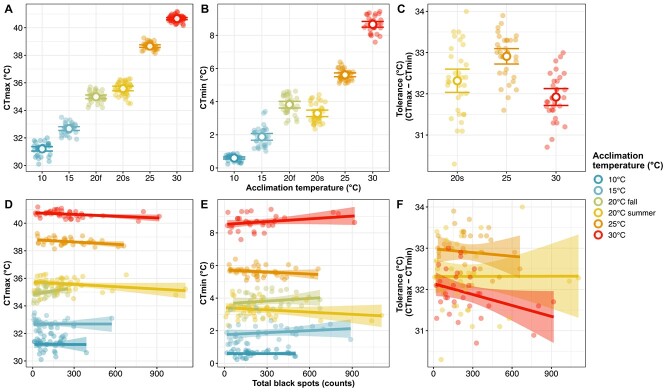
Thermal traits of pumpkinseed across acclimation temperatures and the influence of total black spots. (**A**) CT_max_, (B) CT_min_ and (C) thermal tolerance (CT_max_ - CT_min_) of fish acclimated at 10, 15 and 20°C in the fall and at 20, 25 and 30°C in the summer. Each coloured circle represents an individual, larger white circles represent the means of the treatment and error bars show the 95% confidence interval. Linear regressions estimating the effect of total parasite density on (D) CT_max_, (E) CT_min_ and (F) tolerance are shown for each acclimation treatment with dots as individuals and the shaded zone as the 95% confidence interval for the regression.

Across acclimation temperature treatments (10, 15, 20, 25, 30°C), CT_max_ ranged from 31.20°C ± 0.46 to 40.66°C ± 0.23 (Fig. 2A) and CT_min_ ranged from 0.60°C ± 0.18 to 8.66°C± 0.47 (Fig. 2B). Based on the average CT_max_ value of each group, CT_max_ increased on average by 0.47°C for each degree through acclimation from 10 to 30°C (9.46°C difference).

Following model selection for all thermal traits, CT_max_ was best predicted by black spots, bass tapeworms, yellow grubs, fish mass and acclimation temperature, with no interactions (CT_max_ Model 1, Supplementary Table S1A, AIC = 199.15). Acclimation temperature (R^2^ = 0.99, *F*(5,205) = 2790.90, *P* < 0.001, Table 1 and Fig. 2A) and black spot count (R^2^ = 0.99, *F*(1,205) = 7.94, *P* = 0.005, Table 1 and Fig. 2D)) were significantly related to CT_max_. CT_max_ was higher with increasing acclimation temperature: significant differences between the means of all acclimation groups were found (Tukey’s *post hoc* test (*P* < 0.001), Supplementary Table S1B). Conversely, black spot count was negatively related to CT_max_ (β = −0.0005, *t*-value = −2.82, *P* = 0.005, Supplementary Table S1C). Each black spot decreases CT_max_ by 0.0005°C: i.e. a fish with 1000 black spots has an estimated CT_max_ 0.5°C lower compared to a fish with no black spots.

CT_min_ decreased on average by 0.40°C for each degree from 30 to 10°C (8.06°C difference). CT_min_ was best predicted by a combination of black spots, bass tapeworms, yellow grubs, fish mass, acclimation temperature and an interaction between fish mass and acclimation temperature (CT_min_ Model 1, Supplementary Table S1A, AIC = 275.83). In this model, acclimation temperature (R^2^ = 0.97, *F*(5,185) = 1145.0, *P* < 0.001, Supplementary Table S1B and Fig. 2B) and the interaction between mass and acclimation (R^2^ = 0.97, *F*(5,185) = 4.16, *P* = 0.001, Table 1)) were significantly related to CT_min_. We also found significant differences between the means of each groups (Tukey’s *post hoc* test (*P* ≤ 0.001), Supplementary Table S1B). The only significant interaction between fish mass and acclimation temperature was within the 15°C group (β = 0.105, *t*-value =3.41, *P* = 0.0008).

The best model to explain thermal tolerance (the difference between CT_max_ and CT_min_ for the same individual) included black spots, bass tapeworms, yellow grubs, fish mass and acclimation temperature, with no interaction, (Tolerance Model 1, Supplementary Table S1A, AIC = 210.53). Only acclimation temperature had a significant effect on predicting thermal tolerance (R^2^ = 0.26, *F*(2,93) = 16.19, *P* < 0.001, Supplementary Table S1B and Fig. 2C) and we found significant differences between the 20°C and 25°C groups (*P* = 0.0011), the 25°C and 30°C (*P* < 0.0001) groups, but not between the 20°C and 30°C acclimated fish (*P* = 0.099) (Supplementary Table S1B).

### Influence of parasites on survival between acclimation groups

**Figure 3:**
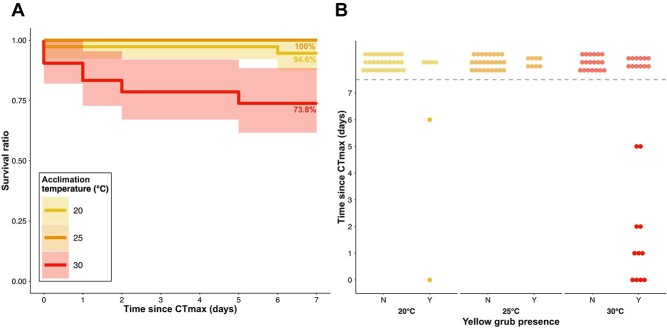
Survival of pumpkinseed in the week following a CT_max_ test across three temperatures (20, 25 and 30°C) and the influence of yellow grub trematodes. (**A**) Kaplan–Meier survival curves for *L. gibbosus* survival post-CT_max_ for three acclimation temperatures including the number of surviving fish per day. (**B**) Mortality events at three acclimation temperatures according the yellow grub presence (Y) or absence (N). The shaded zone represents 95% confidence intervals in (A) and dots represent individuals in (B). Dots above the dotted line represent fish that survived the 7-day period following a CT_max_ trial. Although the model considered the parasites number as a count, they are represented here as a binomial variable for visual purposes.

Mortality following CT_max_ was higher in the 30°C group, as only 73.8% (31/42) of fish survived the week, as opposed to 94.6% (35/37) and 100% (34/34) for the 20°C and 25°C groups, respectively (Fig. 3). Although survival ratio seemed lower at warmer acclimation temperatures (Fig. 3A), as shown by its HR > 1, acclimation was not significant in explaining survival in our best fitting model (coxph model: Acclimation; HR = 1.17, *P* = 0.0896, Table 2). The amount of yellow grubs, on the other hand, was significant in explaining survival through time (coxph model: Yellow grubs; HR = 2.10, *P* = 5.55e-06, Table 2), where an increase in yellow grubs leads to a decrease in survival probability following a CT_max_ trial (Fig. 3B), shown by the HR > 1.

## Discussion

We investigated the relationships amongst thermal tolerance traits, parasites and acclimation to four ecologically relevant temperatures and one future climate change scenario in wild-caught pumpkinseed. We were also able to explore relationships between survival, infection and acclimation temperature following an acute thermal event. As predicted, acclimation temperature affected all thermal tolerance traits measured: CT_max_ was higher for fish acclimated at 30°C, CT_min_ was lowest when fish were at 10°C and thermal tolerance was maximized at 25°C. Interestingly, higher black spot load was related to a lower CT_max_, and survival decreased for individuals infected with yellow grubs after undergoing a CT_max_ trial. However, parasite load did not explain variation in CT_min_ nor thermal tolerance.

**Table 2:** Results of the best fit coxph model estimating the relationships between survival and parasites, fish mass and acclimation temperature

**Predictor**	**HR**	**95% CI**	***P*-value**
Black spots	1.00	1.00, 1.00	0.524
**Yellow grubs**	**2.10**	**1.52, 2.89**	**5.55e-06**
Bass tapeworms	0.97	0.90, 1.04	0.376
Fish mass	0.98	0.74, 1.30	0.892
Acclimation	1.17	0.98, 1.39	0.0896

In bold are the predictors with a significant effect on survival.

CI: Confidence intervals; HR: Hazard ratio.

Our results align with other thermal tolerance studies on pumpkinseed. Under similar acclimation treatments, pumpkinseed collected from ﻿Rice Lake and the Otonabee River (Ontario, Canada) had an average CT_max_ of ~30 and 39.5°C when acclimated at 10 and 30°C, respectively (Rooke *et al.,* 2017), compared to our values of 31.2°C and 40.7°C. The small discrepancy in values is possibly explained by their slower ramping rate of 1°C every 15 min (0.066°C min^−1^). Fishes exposed to faster rates during trials generally have a higher CT_max_ (Illing *et al.,* 2020; Leong *et al.,* 2022). However, not all fishes follow this pattern as white sturgeons (*Acipenser transmontanus*) heated at slow rates showed a higher CT_max_ than when heated at faster rates, suggesting a fast potential for acclimation (Penman *et al.,* 2023). Conversely, CT_max_ in zebrafish measured during slow (0.025°C min^−1^) and fast (0.3°C min^−1^) ramping rates were correlated (Åsheim *et al.,* 2020). The slow thermal ramping used by the authors resembles natural rates of water warming during heat waves experienced in Lake Cromwell. Indeed, temperatures in the littoral zone where our sunfish were collected increased from 24.66 to 27.63°C in 2 h (0.025°C min^−1^) during a heat wave in August (Supplementary Fig. S1). Thus, although we might expect lower absolute CT_max_ temperatures in fish experiencing more natural ramping rates in the wild, our laboratory results likely reflect relative differences in an individual’s upper thermal tolerance measured using fast ramping rates.

Across all acclimation temperatures, we found that CT_max_ was lower in fish that had a high intensity of black spot metacercaria. Our results are consistent with Lutterschmidt *et al.,* 2007, which found that two centrarchid species (*L. macrochirus* and *L. megalotis*) highly infected by trematodes had lower CT_max_ than conspecifics with low parasite intensity (~32°C vs 35.5°C, respectively). Early stages of infection with black spot trigger an immune response in their hosts, which can be energetically demanding (Lemly and Esch, 1984), and potentially detrimental to upper thermal tolerance. The mechanisms underlying this relationship need to be further explored.

The average CT_min_ of our pumpkinseed acclimated at 15°C (1.9°C) was similar to those in Becker *et al.* (1977): 1.7°C. However, we did not find any relationship between parasite load and lower thermal tolerance. Few studies investigate CT_min_ in temperate populations, and even fewer have directly evaluated whether parasites affect CT_min_ (Hockett and Mundahl, 1989). As average temperatures are rising and winters become shorter (Wang *et al.,* 2021), temperate fishes may become more sensitive to acute cold shocks. This can be illustrated by the stark contrast in the average CT_min_ of fish acclimated at 20°C (3.3°C), compared to those at 25°C (7.1) or 30°C (8.7), as the difference in lower thermal tolerance between 25 and 30 is only 1.6°C, whilst it is 3.8°C between 20 and 25. In the context of climate change where average water temperatures and cold shocks are expected to increase (Reid *et al.,* 2022), temperate fishes could be at risk to extreme events due to the rapid loss of lower thermal tolerance at warmer acclimation temperatures. Similar to CT_max_, we predicted that parasite infection would be related to increased CT_min_. Indeed, Lemly and Esch (1984) found that high intensities of black spot metacercaria decrease bluegill (*L. macrochirus*) lipid content, leading to lower survival rates in overwintering fish. The fact that we found no relationship between parasite load and CT_min_ could indicate that the underlying mechanisms of CT_max_ and CT_min_ are differentially affected by parasites: infection may constrain upper thermal tolerance acutely, but limit lower thermal tolerance over longer time periods, such as during overwintering. There is a need to further explore the links between cold tolerance, overwintering survival and infection.

To our knowledge, no study has measured CT_max_ and CT_min_ on the same individuals to obtain thermal tolerance values for pumpkinseed. Here, for three summer acclimation temperatures (20, 25, 30°C) we found that thermal tolerance was highest at 25°C and lowest at 30°C. Although CT_max_ is maximized at 30°C, individuals may be reaching a hard ceiling where acclimation to warmer temperatures will no longer increase CT_max_, possibly limiting their adaptability in a warming climate (Sandblom *et al.,* 2016). Indeed, a simultaneous faster increase in CT_min_ resulted in a smaller thermal tolerance for fish in this acclimation group. Interestingly, the higher thermal tolerance at 25°C suggests that this temperature provides pumpkinseed with a wide thermal window and may be optimal for activities such as growth and reproduction. Exposure to warmer temperatures such as at 30°C may constrain aerobic scope (Jutfelt *et al.,* 2021) or induce a stress response in individuals (Alfonso *et al.,* 2021) as our populations do not typically reach temperatures >28°C in the summer. Although we expected parasites to constrain thermal tolerance across all temperatures, we found no significant relationship between infection and thermal tolerance, which supports the idea that the underlying mechanisms constraining upper and lower thermal tolerance could be affected in different ways by parasite infection.

Although CT_max_ protocols should be non-lethal, most studies only assess survival after 1 h (Ford and Beitinger, 2005) or 24 h (Cowan *et al.,* 2023; Stewart *et al.,* 2023), or omit to mention the survival assessment period (Bennett and Beitinger, 1997; Morgan *et al.,* 2018). Temperatures in the wild can reach or approach an individual’s CT_max_ during massive heat waves (Desforges *et al.,* 2023), and consequences are likely felt over multiple days (Genin *et al.,* 2020; Alfonso *et al.,* 2021). Our results suggest that mortality can occur multiple days after a CT_max_ trial: death occurred at 1, 2, 3 and 6 days post-trial for fish acclimated at 30°C and after 1 and 6 days in the 20°C group. The difference in survival rates across temperatures was best explained by the presence of yellow grubs found in fish gills, where infected fish acclimated at 30°C seemed to have lower survival rates. Although the ecology and life cycle of the yellow grub is well understood (Wang *et al.,* 2017), research on the physiological effects of infection on hosts remains scarce. Considering these parasites target metabolically active organs, such as the heart, gills or muscles, we suggest infection with this parasite imposes an additional toll for recovery following an acute thermal challenge for individuals acclimated to temperatures that are stressful (Marcogliese, 2008). Indeed, parasites have been linked to reduced survival following heat stress in coho salmon (﻿*Oncorhynchus kisutch*) (Teffer *et al.,* 2019), mosquito fish (*Gambusia affinis*) (Granath and Esch, 1983) and three-spined stickleback (*Gasterosteus aculeatus*) (Wegner *et al.,* 2008). Our results suggest that summerkill events caused by heat wave events, which are expected to increase more than fourfold in the northern hemisphere, could impose considerable risk to infected fish (Meehl and Tebaldi, 2004; Seneviratne *et al.,* 2014).

## Conclusions

Our study provides novel information on CT_min_ and thermal tolerance of this species, which is scarce, as well as highlights the need to consider survival when conducting thermal tolerance studies in the lab, as effects may be felt multiple days later. As certain parasites seem to have differential effects, omitting to consider them could indicate that acclimation temperature was the only determining factor explaining survival and thermal tolerance. As we are currently facing exceedingly high amounts of heat waves, fish populations are at risk, and summer die-offs could be expected, highlighting the need to start incorporating parasite load and longer survival assessment in studies on wild fish populations.

## Supplementary Material

Web_Material_coae015

## Data Availability

Data presented in this study are available on figshare: https://figshare.com/articles/dataset/DeBonville_ConPhys2024_csv/25134665. (De Bonville *et al.,* 2024).
