# Supplementary material for: Thermal tolerance and survival are modulated by a natural gradient of infection in differentially acclimated hosts
Source: Conserv Physiol. 2024 Apr 15;12(1):coae015. doi: 10.1093/conphys/coae015 (PMC11020239; doi:10.1093/conphys/coae015)
Supplement: Web_Material_coae015 [file web_material_coae015.zip › DeBonville_Supporting_Information clean.pdf]

SUPPORTING INFORMATION

For : ***Thermal tolerance and survival are modulated by a natural gradient of infection in differentially acclimated hosts***

Figure S1. Lake Cromwell temperature readings (°C) taken with a HOBO logger (HOBO Pendant® MX2201) placed 1 meter below water surface. Data was collected from 2021-06-13 to 2022-08-15 every 10 min. Dashed lines represent the acclimation temperatures chosen for this study. The red inverted triangle represents the warmest temperature recorded (27.97°C) on 2021-08-22.

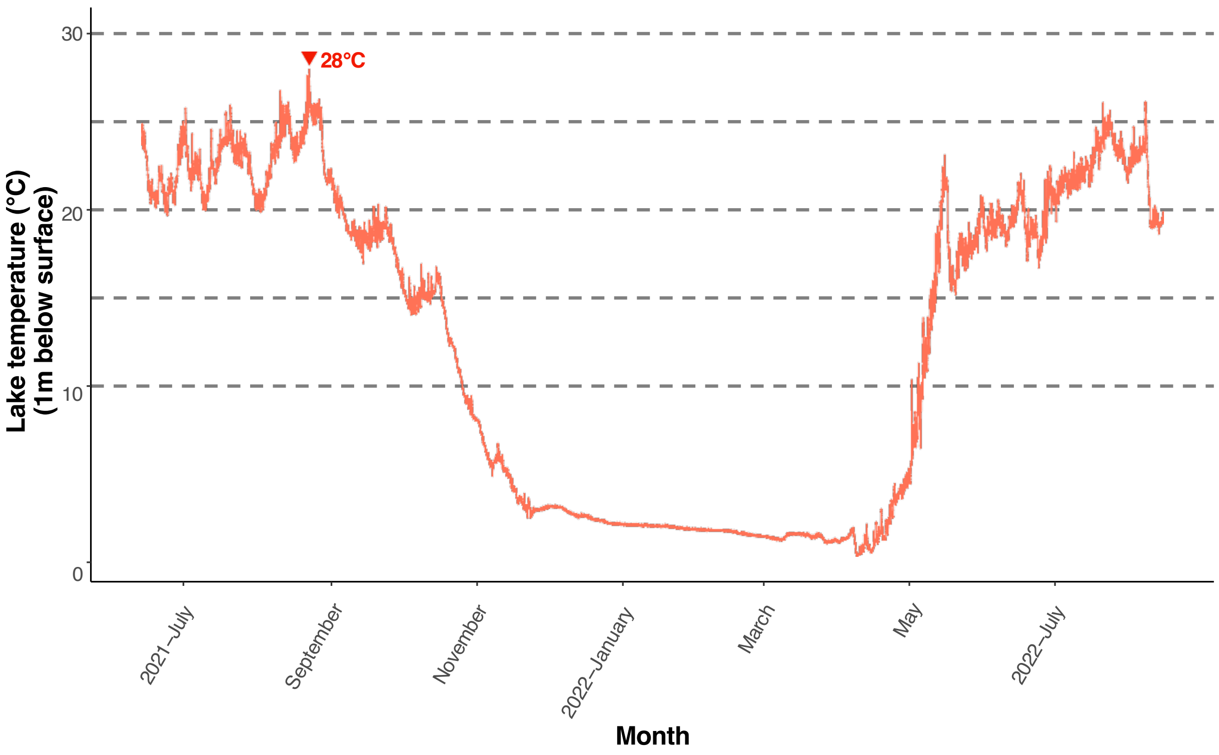

Figure S2. Temperature curves for 600L living stream tanks housing fish in A) Fall 2020, B) Summer 2021 and C) Fall 2021. Temperature was recorded and logged every 10 minute interval in the middle of the tank by HOBO loggers from the moment fish were transferred to the tank until the last CT_{max} or CT_{min} trial. The shaded area behind the lines represent the acclimation period (3 weeks in A and B and 4 weeks in B to account for the week between CT_{max} and CT_{min}). Dashed lines represent the average of a group during the acclimation period while the shaded area also represent the standard deviation.

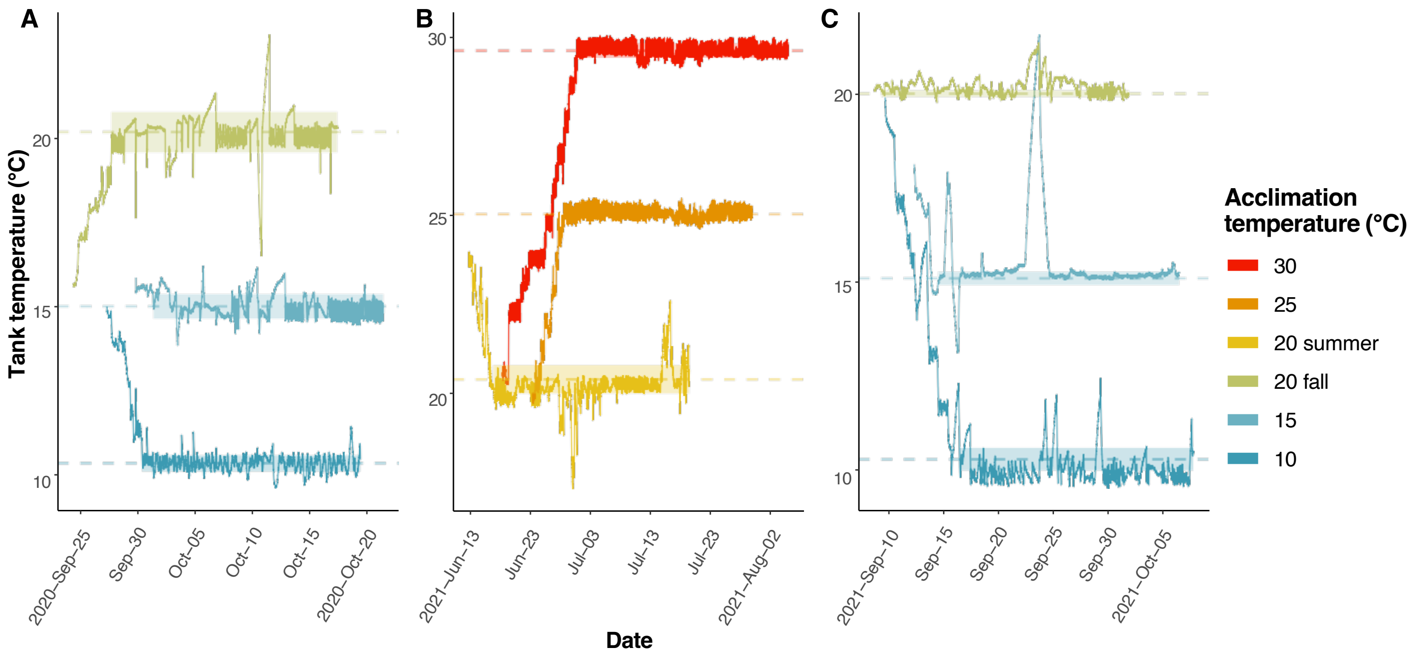

Figure S3. CT_{max}/CT_{min} setup photograph (A) and diagram (B). The setup consists of two tanks where water is continuously pumped from the heating/cooling sump (C) to the testing arena (D). Water flows out of the testing tank through two pumps (E) and after being warmed or cooled, is pumped back into the testing arena from the top. Water in the heating tank is warmed at an average rate of 0.26 ± 0.04 °C $min^{-1}$with coil heaters or cooled at an average rate of -0.3 ± 0.1 °C min^{-1}. Temperature is continuously logged in the middle of the testing arena with a HOBO temperature logger (HOBO® MX2303, USA).

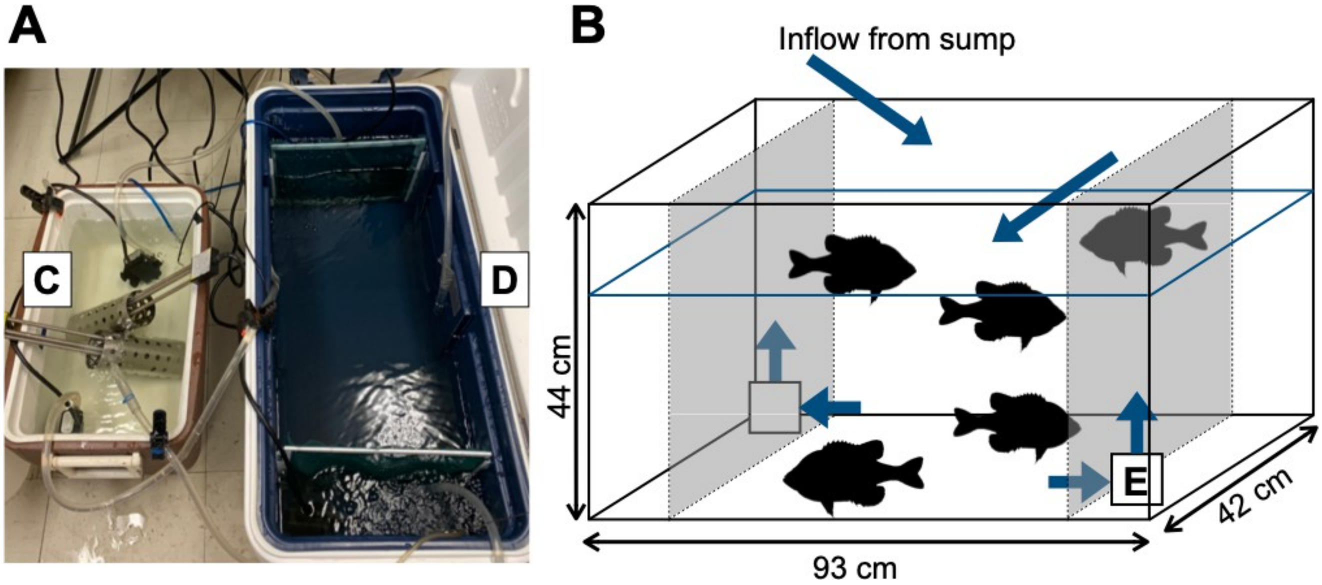

Figure S4 : Thermal ramping curves during trials for CT_{max} (A) and CT_{min} (B) separated by acclimation temperature (color) and by the trial # (linetype). We conducted 2-3 trials at each acclimation temperature. Temperature was logged in the middle of the experimental arena every 2 seconds by a HOBO logger (2020 logger : HOBO® MX2203, 2021 logger : HOBO® MX2303) at the start of the trial (0 minutes) until the last fish reached LOE.

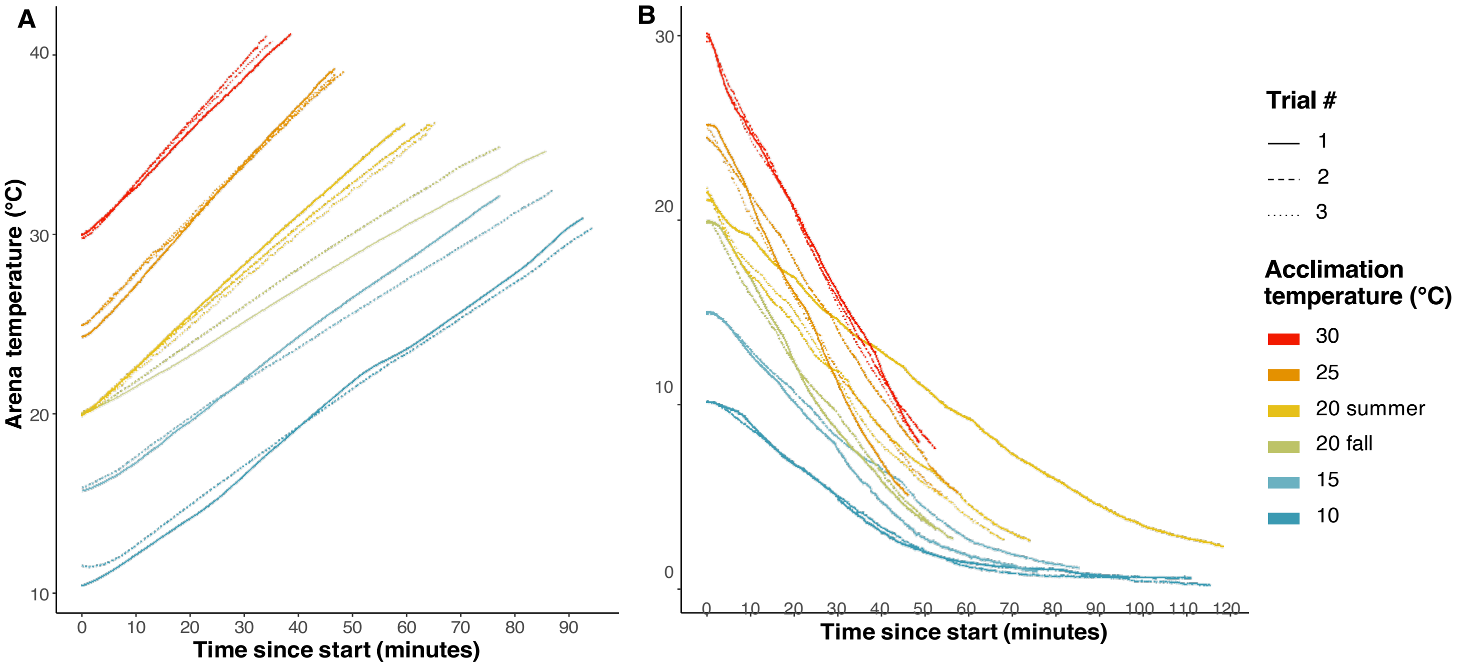

Figure S5 : Ramping rate confirmation trials (0.3°C min^{-1}) in three anesthetized fish of different size which had a Type K Thermocouple inserted in the deep dorsal muscle. The red line represents temperature readings in fish muscle (Type K, RS PRO) and the blue line shows temperature measures in water (HOBO® MX2203). The differences in the intercept of the slope is explained by the delay of readings of the plastic encased water logger (response time : 7 minutes). Ramping rates for the three trials were : mean ± s.d.: Fish = 0.243 ± 0.003 °C min^{-1}; Water = 0.235 ± 0.023 °C min^{-1}).

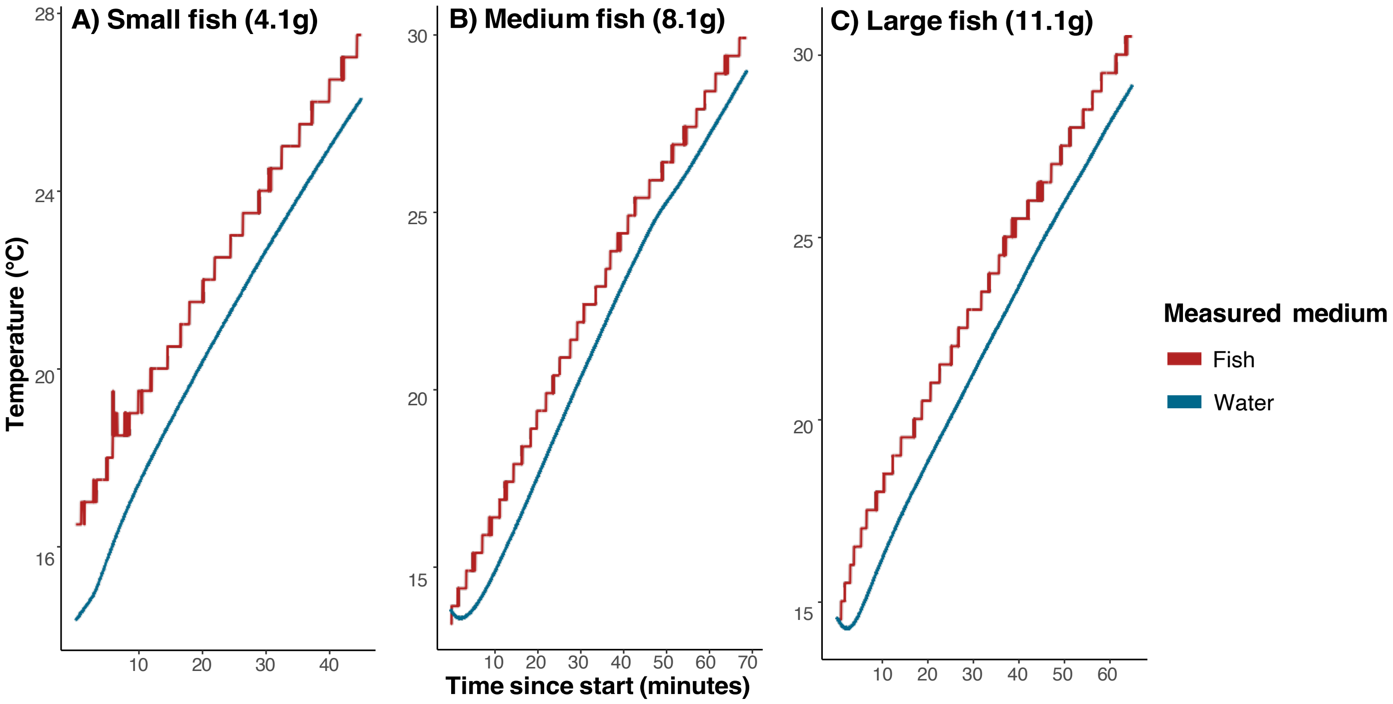

Figure S6 : Comparison of black spot counts on the left and right side of *Lepomis gibbosus* caught in Lake Cromwell in 2022 for a separate project (n=89) including a 1:1 line. Console lines show results from statistical tests.

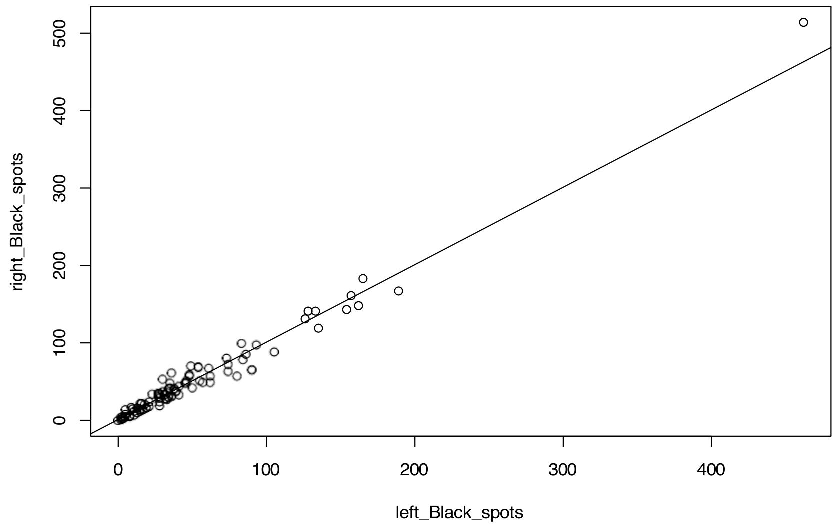

Shapiro-Wilk normality test
W = 0.92495
p-value = 7.039e-05

Wilcoxon signed rank test with continuity correction
V = 1660.5
p-value = 0.2838

Figure S7. Schoenfeld tests for each predictor used in the final coxph survival model (Global *p* – value : 0.1189).

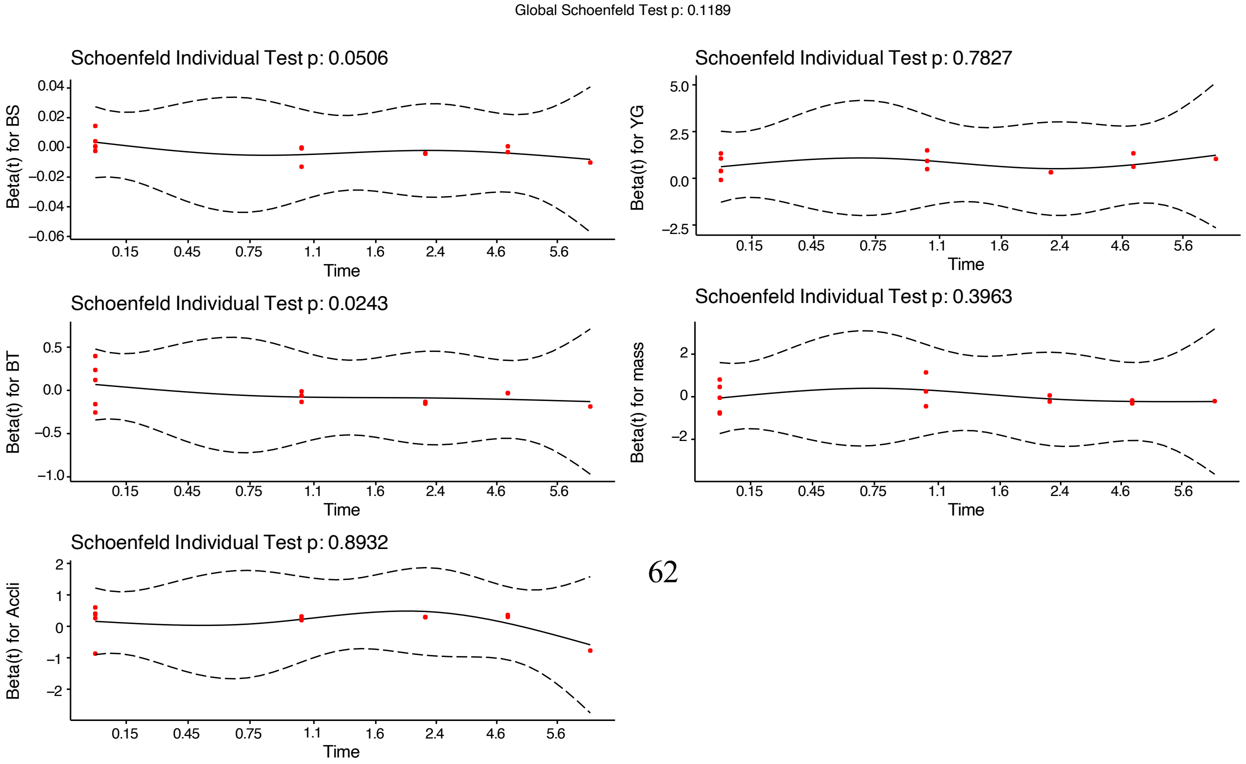

The assumptions of proportional hazard were tested using the cox.zph() and visually observed by plotting the Schoenfeld residuals with the coxzph() function from the survminer package and validating that all predictors were encompassed by the 95% confidence interval lines. The martingale residuals were plotted against covariates to confirm the nonlinearity assumption and influential cases were negligeable when observing residuals of the model (all dfbetas <1).

Table S1A. Summary of Akaike's Information Criterion (AIC) models to find models with the best fit to predict thermal traits (CT_{max}, CT_{min} and tolerance) of pumpkinseed acclimated to multiple temperature treatments. *BS* is the amount of black spots of the fish. *BT* is the number of bass tapeworms in the fish. *YG* is the number of yellow grubs in the fish. F*ish mass* represents the corrected fish mass (parasite mass removed) and *Acclimation* corresponds to the thermal acclimation treatment.

Trait	Model	d.f.	AICc	DAic	weight	Cw	R^2_{aj}
CT_{max}	**1. *BS + BT + YG + Fish mass + Acclimation***	**11**	**199.15**	**0.00**	**0.57**	**0.57**	**0.99**
	2. *BS + BT + YG + Acclimation*	10	200.47	1.32	0.29	0.86	0.99
	3. *BS + BT + YG + Fish mass + Acclimation + BT : Acclimation*	16	203.44	4.29	0.07	0.93	0.99
	4. *BS + BT + YG + Fish mass + Acclimation + BS : Acclimation*	16	204.13	4.98	0.05	0.98	0.99
CT_{min}	**1. *BS + BT + YG + Fish mass + Acclimation + Fish mass : Acclimation***	**16**	**275.83**	**0.00**	**0.97**	**0.97**	**0.97**
	2. *BS + BT + YG + Acclimation*	10	283.86	8.03	0.02	0.99	0.97
	3. *BS + BT + YG + Fish mass + Acclimation*	11	285.57	9.75	0.01	1.00	0.97
	4. *BS + BT + YG + Fish mass + Acclimation+ BS : Acclimation*	16	289.42	13.59	0.00	1.00	0.97
Tolerance	**1. *BS + BT + YG + Fish mass + Acclimation***	**8**	**210.53**	**0.00**	**0.38**	**0.38**	**0.26**
	2. *BS + BT + YG + Acclimation*	7	210.57	0.04	0.37	0.75	0.25
	3. *BS + BT + YG + Fish mass + Acclimation+ YG : Acclimation*	10	213.57	3.04	0.08	0.83	0.26
	4. *BS + BT + YG + Fish mass + Acclimation+ BS : Acclimation*	10	213.76	3.22	0.08	0.91	0.26

Table S1B. Post-hoc contrasts of least square means comparing acclimation groups for each best model chosen for each response variable in table S1A (Confidence level used : 0.95)

Response	Fixed effects	Estimate	SE	df	t.ratio	p-value
CT_{max}	*10 – 15*	-1.497	0.0918	205	-16.302	<.0001
(n=215)	*10 – 20F*	-3.775	0.0901	205	-41.889	<.0001
	10 – 20S	-4.420	0.0919	205	-48.095	<.0001
	10 – 25	-7.519	0.0939	205	-80.093	<.0001
	10 – 30	-9.495	0.0928	205	-102.332	<.0001
	15 – 20F	-2.277	0.0910	205	-25.034	<.0001
	15 – 20S	-2.923	0.0945	205	-30.919	<.0001
	15 – 25	-6.021	0.0952	205	-63.267	<.0001
	15 – 30	-7.998	0.0936	205	-85.476	<.0001
	20F – 20S	-0.645	0.0932	205	-6.926	<.0001
	20F – 25	-3.744	0.0941	205	-39.775	<.0001
	20F – 30	-5.721	0.0935	205	-61.196	<.0001
	20S – 25	-3.099	0.0902	205	-34.342	<.0001
	20S – 30	-5.076	0.0884	205	-57.409	<.0001
	25 – 30	-1.977	0.0905	205	-21.846	<.0001
CT_{min}	*10 – 15*	-1.177	0.119	185	-9.889	<.0001
(n=200)	*10 – 20F*	-3.185	0.121	185	-26.397	<.0001
	10 – 20S	-2.649	0.119	185	-22.336	<.0001
	10 – 25	-4.998	0.123	185	-40.797	<.0001
	10 – 30	-7.971	0.131	185	-61.014	<.0001
	15 – 20F	-2.008	0.114	185	-17.548	<.0001
	15 – 20S	-1.472	0.113	185	-13.066	<.0001
	15 – 25	-3.821	0.118	185	-32.358	<.0001
	15 – 30	-6.793	0.127	185	-53.498	<.0001
	20F – 20S	0.536	0.112	185	4.778	0.0001
	20F – 25	-1.814	0.118	185	-15.389	<.0001
	20F – 30	-4.786	0.128	185	-37.383	<.0001
	20S – 25	-2.349	0.117	185	-20.088	<.0001
	20S – 30	-5.322	0.126	185	-42.222	<.0001
	25 – 30	-2.972	0.132	185	-22.527	<.0001
Tolerance	*20S - 25*	*-0.606*	*0.165*	**93**	**-3.684**	**0.0011**
($CT_{max} – CT_{min}$)	*20S - 30*	0.351	0.168	93	2.082	0.0990
(n=100)	*25 - 30*	0.957	0.171	93	5.594	*<.0001*

Table S1C. Relationship between thermal traits (CT_{max}, CT_{min}, tolerance) and predictors based on the best model chosen for each response variable in table S1A. The 10°C treatment is used as the reference factor level (i.e. intercept) for the CT_{max} and CT_{min} model while the 20°C treatment is used for the tolerance model. Significant effects and their *p-value* are shown in bold.

Response	Fixed effect	Coefficient (β)	Std. error	t-value	*P*	R^2_{aj}
CT_{max} (n=215)	***Intercept***	31.1264669	0.0989393	314.602	**< 2e-16**	0.987
	BS	-0.0004524	0.0001605	-2.819	**0.0053**	
	BT	-0.0006775	0.0009992	-0.678	0.4985	
	YG	0.0125709	0.0289903	0.434	0.6650	
	Fish mass	0.0195192	0.0105789	1.845	0.0665	
	Accli15	1.4971899	0.0918394	16.302	**< 2e-16**	
	Accli20F	3.7746201	0.0901092	41.889	**< 2e-16**	
	Accli20S	4.4197816	0.0918965	48.095	**<2e-16**	
	Accli25	7.5186714	0.0938739	80.093	**< 2e-16**	
	Accli30	9.4953644	0.0927895	102.332	**< 2e-16**	
CT_{min} (n=200)	***Intercept***	0.7883322	0.2408263	3.273	**0.001268**	0.969
	BS	0.0001118	0.0001709	0.654	0.513677	
	BT	-0.0015342	0.0013632	-1.125	0.261866	
	YG	0.0137210	0.0300561	0.457	0.648557	
	Fish mass	-0.0187483	0.0219315	-0.855	0.393736	
	Accli15	0.2631217	0.3231505	0.814	0.416555	
	Accli20F	2.8524469	0.3356739	8.498	**6.30e-15**	
	Accli20S	2.9222318	0.3408939	8.572	**3.97e-15**	
	Accli25	4.9021666	0.3420598	14.331	**< 2e-16**	
	Accli30	8.1034268	0.3681935	22.009	**< 2e-16**	
	Fish mass * 15	0.1045397	0.0306526	3.410	**0.000796**	
	*Fish mass * 20F*	0.0380002	0.0329932	1.152	0.250905	
	*Fish mass * 20S*	-0.0312621	0.0351465	-0.889	0.374901	
	*Fish mass * 25*	0.0110035	0.0371992	0.296	0.767716	
	*Fish mass * 30*	-0.0151974	0.0427741	-0.355	0.722775	
Tolerance (CT_{max} – CT_{min}) (n=100)	***Intercept***	32.0888905	0.2439488	131.539	**< 2e-16**	0.263
	BS	-0.0004549	0.0003224	-1.411	0.161589	
	BT	0.0007207	0.0026036	0.277	0.782529	
	YG	-0.0086165	0.0722427	-0.119	0.905317	
	Fish mass	0.0398005	0.0264782	1.503	0.136190	
	Accli25	0.6063314	0.1645747	3.684	**0.000385**	
	Accli30	-0.3506683	0.1683939	-2.082	**0.040049**	

Table S2. Survival model selection with analysis of deviance table using the likelihood ratio test (LRT) comparing fits of multiple coxph models (*BS* : Black spots, *YG* : Yellow grubs, *BT* : Bass tapeworms, Accli : Acclimation temperature, mass : Fish corrected mass). The model in bold represents the best fitting model.

Model	d.f.	LogLik	χ^2	p-value
2. *(Time, Status) ~ BS + YG + BT + mass * Accli*	6	-45.05		
3. *(Time, Status)* ~ BS + YG + BT * Accli + mass	6	-42.17	5.74	<0.001
4. *(Time, Status)* ~ BS + YG * Accli + BT + mass	6	-44.99	5.62	<0.001
5. *(Time, Status)* ~ BS * Accli + YG + BT + mass	6	-44.78	0.41	<0.001
**6. *(Time, Status)* ~ BS + YG + BT + mass + Accli**	**5**	**-45.31**	**1.06**	**0.303**
7. *(Time, Status)* ~ BS + YG + BT + Accli	4	-45.32	0.02	0.892
9. *(Time, Status)* ~ 1 (Null model)	0	-60.74	30.84	<0.001

Coxph model selection was conducted by including all predictors in the model and reducing to less complicated models, dropping the non-significant interactions and other predictors (such as fish mass), to choose the model with the lowest log likelihood (Cortese et al., 2021). Although the more complex models including interactions generally had a higher Loglik, no interaction was significant, so they were removed and the next best model was chosen, which only included all the parasites, fish mass and acclimation treatment (LogLik : -45.31, p =0.303). This model respected all assumptions and displayed a significant likelihood ratio test (LRT = 30.86 on 5 df, p = 1e-05).
